# Experts’ moral views on gene drive technologies: a qualitative interview study

**DOI:** 10.1186/s12910-021-00588-5

**Published:** 2021-03-08

**Authors:** N. de Graeff, Karin R. Jongsma, Annelien L. Bredenoord

**Affiliations:** Department of Medical Humanities, Julius Center for Health Sciences and Primary Care, University Medical Center Utrecht, Utrecht University, Utrecht, The Netherlands

**Keywords:** Ethics, Qualitative research, Gene editing, Gene drives

## Abstract

**Background:**

Gene drive technologies (GDTs) promote the rapid spread of a particular genetic element within a population of non-human organisms. Potential applications of GDTs include the control of insect vectors, invasive species and agricultural pests. Whether, and if so, under what conditions, GDTs should be deployed is hotly debated. Although broad stances in this debate have been described, the convictions that inform the moral views of the experts shaping these technologies and related policies have not been examined in depth in the academic literature.

**Methods:**

In this qualitative study, we interviewed GDT experts (n = 33) from different disciplines to identify and better understand their moral views regarding these technologies. The pseudonymized transcripts were analyzed thematically.

**Results:**

The respondents’ moral views were principally influenced by their attitudes towards (1) the uncertainty related to GDTs; (2) the alternatives to which they should be compared; and (3) the role humans should have in nature. Respondents agreed there is epistemic uncertainty related to GDTs, identified similar knowledge gaps, and stressed the importance of realistic expectations in discussions on GDTs. They disagreed about whether uncertainty provides a rationale to refrain from field trials (‘risks of intervention’ stance) or to proceed with phased testing to obtain more knowledge given the harms of the status quo (‘risks of non-intervention’ stance). With regards to alternatives to tackle vector-borne diseases, invasive species and agricultural pests, respondents disagreed about which alternatives should be considered (un)feasible and (in)sufficiently explored: conventional strategies (‘downstream solutions’ stance) or systematic changes to health care, political and agricultural systems (‘upstream solutions’ stance). Finally, respondents held different views on nature and whether the use of GDTs is compatible with humans’ role in nature (‘interference’ stance) or not (‘non-interference stance’).

**Conclusions:**

This interview study helps to disentangle the debate on GDTs by providing a better understanding of the moral views of GDT experts. The obtained insights provide valuable stepping-stones for a constructive debate about underlying value conflicts and call attention to topics that deserve further (normative) reflection. Further evaluation of these issues can facilitate the debate on and responsible development of GDTs.

**Supplementary Information:**

The online version contains supplementary material available at 10.1186/s12910-021-00588-5.

## Background

Gene drive technologies (GDTs) are genome editing technologies that promote the rapid, progressive spread of a particular genetic element within a population of non-human organisms. Whereas a given gene is passed on to approximately half of an organism’s offspring in normal Mendelian inheritance, gene drives can promote the biased inheritance of a particular gene, so that this gene is passed on to most or even all of an organism’s offspring.^1^ If organisms reproduce quickly, the edited trait can consequently spread rapidly and permanently across the population [1]. In the past few years, GDTs have advanced substantially, from a largely theoretical proposal to proof-of-concept experiments in various organisms [2, 3]. While a number of natural and synthetic gene drive systems based on different molecular mechanisms exist [4], the gene-editing tool CRISPR/Cas9 (Clustered Regularly Interspaced Palindromic Repeats/CRISPR-associated protein 9) has led to particularly significant advancements in GDTs [2]. Gene drives are now “on the horizon” [1].

GDTs have been proposed as a potential strategy to address several major problems, including the burden of vector-borne diseases such as malaria [5], the agricultural, economic, and environmental damage caused by invasive species [6], and the rise of pesticide and herbicide resistance in agricultural settings [7]. Additionally, GDTs could be used in basic research, for example to construct animal models of human disease [8]. Various types of gene drive designs have been proposed, ranging from self-sustaining gene drives which are designed to spread throughout all populations of a species, to self-limiting or thresholded gene drives that are spatially or temporally limited in their spread [1].

The development and possible use of GDTs has stirred considerable scholarly debate. Major concerns in this debate relate to biosafety and biosecurity issues, including the safeguarding of laboratory experiments with GDTs and potential negative effects on ecosystems due to unintended consequences or misuse of the technology [9, 10]. Several papers have mapped the ‘ethical landscape’ and explored various ethical aspects related to GDTs [11, 12]. Other authors have analyzed specific concerns with regard to these technologies, including objections pertaining to 'playing God' and the presumed intrinsic wrongness of tampering with nature [13], intergenerational equity issues [14] and issues related to decision-making about these technologies [15–17]. Finally, various guidelines [1, 18–21], consensus statements and workshop reports [22–27] on the scientific, ethical, social, legal and policy implications of GDTs have been published.

A key question in the debate on GDTs is whether—and if so, under what conditions—GDTs should be deployed, with different organizations and stakeholders taking diverging stances. On one side of the spectrum, parties stress the potential of GDTs and argue this provides a strong argument to develop these technologies. These organizations and stakeholders mostly advocate a phased testing approach in which GDTs are investigated in a step-wise manner: first in laboratory studies, then in small-scale, confined field experiments, followed by open small-scale releases and finally large-scale field releases [1, 18, 28]. On the other side of the spectrum, others contend these technologies are too risky or ethically impermissible on other grounds, and argue in favor of a moratorium on field applications of GDTs [29–31]. Whilst the stances of particular organizations and stakeholders [3, 22, 32, 33] as well as a range of ethical and governance issues related to GDTs [1, 11–17, 25] have been identified and described in the literature, the convictions that inform the stances of a wide range of GDT experts have not yet been examined in depth.

Qualitative interviews are a valuable method to identify, better understand, and juxtapose people’s moral views; they can improve the understanding of ethical implications of a technology by providing insights into how interviewees view and weigh different ethical aspects [34]. In this study, we therefore aimed to investigate the moral views of gene drive experts working in various disciplines through a qualitative interview study. We considered it particularly relevant to study the moral views of experts that are actively involved in (the debate on) GDTs, as they are likely to shape these technologies and influence related policymaking. Technological development and related policymaking are human processes; they are not neutral, but rather influenced by the attitudes, convictions and values of those that shape these technologies and the debates about them [35, 36]. By providing insight into the moral views of gene drive experts and linking our results to the previously published literature, this analysis intends to facilitate a more informed and reflected debate on these disputed technologies, and in turn hopes to contribute to their responsible development.

## Methods

We performed a qualitative interview study to investigate the moral views of gene drive experts from a variety of disciplines. The study is reported in accordance with the consolidated criteria for reporting qualitative studies (COREQ) [37].

### Participant selection and recruitment

Professionals were considered eligible for inclusion in the study if they had contributed to academic publications and/or policy documents on GDT research and development. Eligible participants were identified through a review of the academic [9] and policy publications on GDTs and through so-called snowball sampling, i.e. based on recommendations by previous participants [38]. Based on such snowball sampling, three professionals with broader expertise on respectively global research ethics, anthrozoology (human-non-human-animal studies), and the ethics of gene-editing technologies were also included given the relevance of these fields for the debate on GDTs. To capture a wide range of perspectives on GDTs, a variety of experts from different professional backgrounds and countries were identified. Potential participants were approached and informed about the set-up of the study by e-mail by NG. Recruitment was ended when saturation was reached, i.e. when subsequent interviews no longer brought up new issues (‘coding saturation’) and the formulated themes were sufficiently understood (‘meaning saturation’) [39].

### Data collection

Semi-structured interviews were conducted by NG (trained qualitative researcher, female, MA, MD, PhD candidate). In 28 out of 33 interviews, there had been no previous contact between the interviewer and the participant beforehand; in 5 out of the 33 interviews, the interviewer and the participant had met each other prior to the interview in research meetings or a research visit. The interview guide for the interviews (see Additional file 1) was based on an analysis of the ethical arguments related to GDTs that were identified in a previous review [9] and in discussions amongst the research team. The interview consisted of open-ended questions related to potential benefits, hazards and risks of GDTs, stakeholder involvement and governance of GDTs. This article reports the interview findings related to what may be classified as the substantive ethical questions, concerns, and implications of GDTs, i.e. those questions, concerns, and implications that relate to “what is right in terms of duties, rights, and values (..) independent of any decision-making procedure” [40] (p. 155).^2^ The semi-structured design of the study ensured consistency in a number of topics to be discussed by all participants, while also allowing participants to bring up or emphasize particular new issues they considered relevant. Interviews were conducted in English or Dutch and either took place at a location chosen by the participant (for 25 of the 33 interviews), or online via a video conferencing platform (for 8 interviews). An intern (female biomedical science student, BSc) listened to 3 interviews. The interviews were audiotaped, transcribed verbatim, and pseudonymized.

### Data analysis

The pseudonymized transcripts were analyzed thematically [41]. An initial coding list was developed based on the topic list, familiarization with the data, and discussion in the research team (NG, KRJ, ALB). Subsequently, NG coded a sample of the transcripts. KRJ critically (re)read this sample of coded transcripts, and the interpretations and suitability of the codes were discussed and compared amongst the research team. The coding list was evaluated and adapted, and all interviews were coded by NG using Nvivo 12 software. The meaning of individual text fragments was determined by interpreting them in the context of the whole interview with the participant in question [42]. In the course of analysis, codes were adapted and additional codes were added to the coding list where necessary. A meaning pattern was identified across the data set, leading to the formulation of interpretative higher order themes. Throughout the process of analysis, the research team went back and forth between the different steps to allow for constant comparison. In the last stage, relevant quotes were selected to illustrate the identified themes.

## Results

Out of the 43 experts that were approached, 33 agreed to participate in the study, 8 were unable to participate and 2 did not respond. A total of 33 semi-structured interviews were conducted between November 2018 and July 2019. The interviews lasted between 49 and 114 min, with an average duration of 69 min. 13 respondents were employed in the United States, 11 in the United Kingdom, 8 in various European countries (Belgium, France, Spain, Switzerland, and the Netherlands) and 1 in Burkina Faso. Interview respondents worked in different disciplines, including the natural sciences (n = 11), philosophy/ethics (n = 9), non-governmental organizations (NGOs; n = 5), policy-making (i.e. professionals working in an organization that is involved in designing policy or regulations for GDTs or that funds gene drive research; n = 5) and various social sciences (n = 3). Those individuals who were working in the natural sciences and affiliated with an NGO (n = 2) were classified as ‘natural scientists’.

Three main themes were identified during the data analysis. The moral views of the respondents were principally influenced by their attitudes towards or convictions about [1] how best to deal with the uncertainty related to GDTs; (2) which alternatives should be weighed and how; and (3) their views on nature and the role humans should have in nature. The tables list representative quotations that were selected to illustrate the identified themes. In the following, we indicate the respondents’ disciplines only if it helps to contextualize their opinions in comparison to respondents with different stances.

### Theme 1: dealing with uncertainty

#### Identifying sources of uncertainty

Many respondents stressed the potential of GDTs, yet at the same time reflected on the epistemic (knowledge-related) uncertainty about whether GDTs will be successful at achieving their intended goals. Similarly, many respondents reflected on the difficulty of accurately predicting the potential negative effects of GDTs.

The respondents identified different, interrelated sources of uncertainty. First, some respondents stressed that the proposed applications of GDTs and particular gene drive designs are based on mathematical modeling and limited proof-of-concept studies and are still being developed in the laboratory (Table 1, Quote 1A). Second and relatedly, some respondents identified knowledge gaps that contributed to epistemic uncertainty. These knowledge gaps related to the efficacy and hazards of GDTs in laboratory and cage experiments, population dynamics and sizes of natural populations in which GDTs may be used, and the roles of these populations in their ecosystems (Table 1, Quote 1B). Third, various respondents (all natural scientists) expressed concerns about the technical hurdles that have been encountered in making stable GDTs (Table 1, Quote 1C), in which the necessary components are expressed at the right time, place and level, without excessive fitness costs or resistance occurring. Similarly, some natural scientists reflected on the difficulty of getting GDTs to work in particular species. Finally, several respondents (all natural scientists) reflected on the complexities involved in translating results from the laboratory to the field; results in the laboratory may differ from results in ecosystems, complicating estimations about the effects of GDTs based on laboratory results (Table 1, Quote 1D).

**Table 1:** Quotations that illustrate theme 1

	(Sub)theme	Quote
1	Dealing with uncertainty	
	*Identifying sources of uncertainty*	
1A	More experimental evidence must be obtained	R1: “Various designs (..) work mathematically. But sometimes biology is different than theory. So these models should be tested experimentally in the laboratory the upcoming years”
1B	Knowledge gaps	R21: “There’s a lot we don’t know right now and there’s much more study that needs to go, that needs to happen before we start releasing gene drives into the environment”
1C	Technical hurdles	R26: “All these proof of principle drives that have been published, they’re (..) very gentle to the genome, which means that they’re easy to show good principals in the lab, but they’re not strong enough to be able to spread robustly once you get them into the wild. And so yes, the issue we’re encountering now is—we know how in theory we should build them—to make them spread strongly in the wild. But there’s just so many engineering hurdles to get that to work, right”
1D	Translation from laboratory to field	R6: “There are so many idealizations in populations genetics models that I would not want to stake a whole lot on them being accurate predictors of what happens when you intervene [in the wild]”
	*Dealing with epistemic uncertainty*	
1E	Epistemic uncertainty as a reason to support a moratorium	R29: “I think in terms of the moratorium scientists are not even at the stage yet of asking the right questions about gene drives, let alone building enough understanding of genes and evolution to release gene drives into the environment”
1F	Epistemic uncertainty as a reason to support phased research in light of the status quo	R12: “(..) the status quo situation we find ourselves in is already attended by significant harms. That’s certainly the case with malaria. (..) [and so] I think we ought to push back a little against this overly precautious approach. And that’s not to say I’m going to absolutely support releasing (..) [a] gene drive organism. But I think in order to make an informed decision about whether we should be doing field trials or more general releases, we really need to know more about what the technology can and can’t do”
1G	Accepting a certain level of epistemic uncertainty	R14: “There are many, many reasons why it might fail in the field (..) but there’s a certain point where we have to say “it’s good enough and we can’t see any obvious reason why it’s going to fail”
1H	Efforts undertaken to study knowledge gaps should be acknowledged	R31: “It never ceases to amaze me that these things are still years away from actual release and yet they’re in the focus of such an intense scrutiny already, and a lot of the questions raised are questions that we’re really trying hard to answer and would not go to the field without answering. But, you know, it is bound to cause confusion with the public that we can’t answer them yet. (..) It’s going to take a while to answer these questions and, in the meantime, the public is getting hit with this uncertainty, uncertainty, uncertainty, and so it’s complicated”
1I	Justifying a ‘leap of faith’	R12: “The big question is going to be when we have to consider potential harms to ecosystems because that’s obviously something that’s quite difficult to model in constrained environments. So that’s going to be the leap of faith at the moment. (..) we’re going to have to again, make a balance to think: what are the kinds of important interests that might justify the leap of faith? (..) My view is that (..) it’s going to depend on the degree to which the benefit (..) plays a central role in either human wellbeing or the wellbeing of other features in our environment, including animals”
	*Importance of setting realistic expectations*	
1J	Overhyping may block further development at a later point	R14: “There’s a genuine risk that we put too much hope and faith in gene drives and that they don’t work very well. (..) people need to have a realistic view of what could happen after a gene drive release. And that we don’t have an expectation that the gene drive is released and it’s the first one and we, you know, are still trying to understand how it might spread, how population dynamics come into it, and migration of the mosquitoes, and seasonal effects. And it spreads for a short while and then fails because something stops it from spreading farther. (..) if these things happen, I don’t think that should be a block to further development”
1K	Overhyping creates a false dichotomy	R32: “This silver bullet narrative is damaging. (..) if you propose something as a silver bullet, then you’re somehow some sort of [curse word] if you decide not to use it if it could be this cure-all. We don’t even know if it is, you know? (..) it promotes this binary discussion of “okay, fine, if you don’t use it you want all these people to die (..) and no, that’s not it. (..) I don’t want children to be dying either, but I also don’t want us to make decisions based on something like, someone’s crazy vision of something that maybe isn’t necessarily true yet. We don’t know if it is”

#### Dealing with epistemic uncertainty

Respondents had different views about the implications of the knowledge gaps and epistemic uncertainty related to GDTs and how this uncertainty should be dealt with. A few respondents (predominantly working within the social sciences and the NGO sector) argued the epistemic uncertainty related to GDTs provided a reason to support a moratorium on applications of GDTs outside the laboratory (Table 1, Quote 1E) (‘risks of intervention’ stance). Other respondents (working in various different disciplines) instead argued that such an approach would itself be harmful given the problematic status quo. According to them, the problems that GDTs aim to tackle are themselves attended by significant harms, and this should be factored into the decision on whether to use GDTs (‘risks of non-intervention’ stance). Rather than categorically refraining from applications of GDTs outside the laboratory, they argued more knowledge needs to be obtained about their (intended and unintended) effects through continued phased research to make an informed decision about whether field trials and more general releases should be allowed (Table 1, Quote 1F). Several respondents of the latter group argued that epistemic uncertainty is inherent to the initial stages of technology development and therefore does not provide an argument against developing and at some point testing these technologies (Quote 1G). One respondent, for instance, stressed that these knowledge gaps and related uncertainty do not provide a reason to put GDTs under intense scrutiny this early in the developmental process (Table 1, Quote 1H). One respondent argued a certain level of uncertainty could be justified for GDT applications that could be beneficial for human wellbeing (Table 1, Quote 1I).

#### Importance of setting realistic expectations

Although respondents thus varied in their assessment of how we should deal with the knowledge gaps and uncertainty related to GDTs, respondents with different views agreed that these knowledge gaps and uncertainties have not received enough attention in public and academic debates on GDTs. Various respondents mentioned that GDTs are regularly overhyped or presented as ‘silver bullets’. Respondents mentioned varying reasons why such overhyping is problematic and potentially harmful. On the one hand, several respondents (all natural scientists) who were in favor of developing GDTs mentioned that such overhyping could lead to unrealistic expectations about the technology, which could stifle further development if a first GDT release did not live up to expectations (Table 1, Quote 1I). On the other hand, several respondents with diverging views about GDTs (and from different disciplines) argued the silver bullet narrative created a false dichotomy in the debate about GDTs, in which employment of a perfectly functioning technology or acceptance of the status quo are presented as the only potential choices and outcomes, whereas the potential choices and outcomes are much more complex and uncertain (Table 1, Quote 1J).

In sum, respondents with different views on GDTs agreed there is epistemic uncertainty related to GDTs and identified similar knowledge gaps that ought to be addressed. Similarly, they agreed—albeit for different reasons—that realistic expectations should be set in the academic and public debates on GDTs: experts that participate in these debates should openly address the uncertainties and complexities involved in estimating the effects of GDTs. What they did not agree about was whether epistemic uncertainty provides a reason to refrain from testing GDTs outside the laboratory (‘risks of intervention’ stance) or rather—given the harms of the status quo—a reason to support phased research (‘risks of non-intervention’ stance). Respondents working in the natural sciences, philosophy/ethics and policy making somewhat more often held the former stance, whereas respondents working in the social sciences and NGO sector more often held the latter stance.

### Theme 2: identifying and weighing alternatives

Although almost all respondents morally evaluated GDTs by comparing them to alternatives, respondents identified and used different alternatives in their comparisons, resulting in different conclusions about the permissibility of GDT applications. These alternatives can broadly be grouped in two categories: ‘downstream’ solutions that comprise conventional strategies to target vector-borne diseases, invasive species and agricultural pests, and ‘upstream’ solutions to these issues that instead comprise systematic changes to global health care, political and agricultural systems.

#### ‘Downstream’ solutions: comparing GDTs to conventional strategies

Many respondents (from different disciplines) compared GDTs with conventional strategies used to target vector-borne diseases, invasive species and agricultural pests. For applications to target vector-borne diseases, these alternatives included strategies such as insecticides, impregnated bed nets, swamp draining and antimalarial medication; for applications to target invasive species, these alternatives included the use of pesticides, poisoning and ecosystem interventions such as introducing predators. Many respondents argued GDTs should be developed and/or used for particular applications if they provide benefits in comparison to conventional strategies that are currently being used. For example, numerous respondents contended that alternative conventional strategies have thus far been inadequate and/or harmful for the environment, other species or humans. For them, the harmfulness (Table 2, Quote 2A) and inadequacy (Table 2, Quote 2B) of these conventional strategies underline the need for an alternative strategy to tackle these problems, and GDTs could be such a strategy that could be used next to conventional approaches (Table 2, Quote 2C).

**Table 2:** Quotations that illustrate theme 2

	(Sub)theme	Quote
2	Identifying and weighing alternatives	
	*Comparing GDTs to conventional strategies*	
2A	Conventional strategies are harmful, underlining the need for an alternative strategy	R18: “Our current [anti-malarial] tools, they do have a negative effect. We treat it as being the status quo and so therefore we don’t measure the negative effects, but every pesticide we use on an environment still has a negative, [whichever] we choose. You should compare like with like, but it’s very infrequent that people compare like to like, we have a much greater fear of the new and the novel, as opposed to the cost that we are having already (..) Let’s not say our [anti-malarial] tools are currently not teratogenic or highly problematic to human health”
2B	Conventional strategies are inadequate, underlining the need for an alternative strategy	R5: “The tools we have are good because they’ve saved lives but they’re not perfect or sufficient. Which is why we need something new and this could be it.”
2C	GDTs are complimentary to conventional strategies	R14: “it doesn’t take anything away from what we are already doing. (..) it adds to all of the different interventions. (..) Even if they were around the corner, even if they got used and even if they were being successfully used, don’t stop the other interventions. You’d be mad to do that”
	*Comparing GDTs to systematic changes to global health, political and agricultural systems*	
2D	Agricultural change is needed to solve the problem of agricultural pests	R11: Like if you want to use [gene drives] in agriculture, what does that mean? Are we going to eliminate pests, so called pests, that actually are there because of the way we have chosen to do agriculture, which has proven to be a real problem for the climate, as well as for biodiversity?”
2E	Health care and political change is needed to target vector-borne disease	R16: “I would say at many places it’s mainly a political thing. If you have like [a] good water system, good hospitals, good access to treatment; that would make that the malaria issue is much less problematic”
	*Exhausting alternatives and feasibility of alternatives*	
2F	Questioning whether conventional strategies have been exhausted	R29: “You can look at countries like Paraguay and a number of other countries that have recently been declared as malaria-free, and there’s a lot of really wonderful studies as to what they did. I mean, there are so many approaches from the policy level to the grassroots level and education; so many different strategies and tactics that all need to be implemented”
2G	Changes in global health care, political and agricultural systems not feasible	R18: “You would need to spend a crazy amount of money in the sites where we work to be able to reach the social determinants of health high enough to stop malaria transmission, it would be huge, it’s unattainable”
2H	Difficulty in deciding what should be taken as a given or as changeable	R6: “This is the very hard thing in this area, is to say what to keep fixed”

#### ‘Upstream’ solutions: comparing GDTs to systematic changes

Some respondents (mostly working within the NGO sector and the social sciences) instead compared GDTs with large-scale changes in our global health care, political and agricultural systems. According to these respondents, these underlying systems produce the problems we are trying to tackle in the first place, and if we do not look for the solution of the problem at that level, we are merely controlling the symptoms rather than the underlying problems. One respondent, for instance, argued agricultural pests are present due to the way in which we have designed our agricultural system, and should correspondingly be addressed by changing this system rather than by developing GDTs (Table 2, Quote 2D). Similarly, another respondent contended that, rather than develop GDTs, we should target vector-borne diseases by improving living conditions and health care facilities in the areas where these diseases are endemic (Table 2, Quote 2E). Correspondingly, as GDTs do not get to the root of the problems they aim to solve, these respondents considered GDTs an undesirable intervention.

#### Exhausting alternatives and feasibility of alternatives

Respondents disagreed with each other about whether the alternatives identified by those with a different view were feasible, and about whether they had been sufficiently explored. On the one hand, some respondents that opposed GDTs and argued in favor of ‘upstream’ solutions also questioned whether the conventional ‘downstream’ strategies to deal with vector-borne diseases and invasive species have been exhausted (Table 2, Quote 2F). On the other hand, some respondents who were open to (applications of) GDTs and considered other ‘downstream’ approaches insufficient or harmful, argued that the systematic ‘upstream’ changes advocated by opponents of GDTs to solve the problems at hand may be desirable, but not feasible. According to these respondents, past efforts and future projections by organizations such as the WHO demonstrate that it is naïve to think that the social determinants of health could be increased to such an extent that malaria transmission could be stopped (Table 2, Quote 2G).

All in all, most respondents morally evaluated GDTs by comparing them to alternatives, yet respondents held very different views on which alternatives should be considered (un)feasible and (in)sufficiently explored, and likewise which aspects of the global health care, political and agricultural systems should reasonably be taken as a given or as changeable. These different views, which may be summarized as ‘downstream solutions’ and ‘upstream solutions’ stances, were based on both empirical convictions about past efforts and future projections, as well as on normative convictions about the permissibility of using technology to solve problems that are (in part) caused or exacerbated by social or political processes. Respondents working within the natural sciences, philosophy/ethics and policy making were somewhat more inclined to have a ‘downstream solutions’ stance, whereas respondents working in the social sciences more often referred to the importance of ‘upstream solutions’. These different stances underline a core feature of disagreement about the moral permissibility of using GDTs.

### Theme 3: the role of humans in nature

Finally, respondents had diverging views on what they considered justifiable interventions in nature, and whether GDTs could be considered a justifiable intervention. In other words, respondents differed in their assessment of what the role of humans in nature should be, and whether it is morally permissible to intervene in wild ecosystems in this way.

#### Assessing the moral permissibility of interventions in nature

Several respondents (none of whom were scientists or policy makers) argued we should not intervene in nature by using GDTs (‘non-interference’ stance). According to these respondents, the natural state of affairs is something that ought to be protected, and that would be disrupted by the use of GDTs. By using GDTs, some of them argued, humans would take up the role of ‘designers’ of nature, and this would be morally impermissible (Table 3, Quote 3A). Several respondents stressed these concerns about the role that humans should have in nature do not just apply to the use of GDTs, but are rather a part of broader concerns about the negative impact of humans on earth. These respondents emphasized that the human relationship to nature is largely skewed towards changing nature, rather than living in balance with nature and trying to preserve the natural state of affairs, and that this is generally undesirable (Table 3, Quote 3B). A few respondents mentioned it could be considered specifically problematic if suppression drives were used to eradicate unwanted populations or species (Table 3, Quote 3C).

**Table 3:** Quotations that illustrate theme 3

	(Sub)theme	Quote
3	The role of humans in nature	
	*Assessing the moral permissibility of interventions in nature*	
3A	We should not take up the role of designers of nature by using GDTs	R30: “[I] have a problem with it, there is this nagging idea, that (..) we have this ability to say in a finite way “we’re changing this organism and we’re going to turn this organism from a vector into some type of benevolent tool for our use”
3B	Concerns about the role of humans in nature are part of broader concerns about the impact of humans on earth	R24: “[There is this] very fuzzy sense that it’s nice to try to preserve the natural state of affairs. (..) [We should preserve] the human relationship to nature and the desire to live with the world rather than always changing the world. (..) We're doing an incredibly bad job of that. There's no balance whatsoever at the moment, and gene drives are, you know, not the main story. The main story is (…) climate change, and total ecosystem disruption, deforestation and pollution. (..) But in so far as you know, we’re talking about the ethics of gene drives (..) I do think about (..) applications in that way”
3C	It would be impermissible to suppress or extinct species that humans consider undesirable	R24: “There’s a sense in which gene drives can be thought of as extinction technologies. They’re getting rid of something you don’t want, either the whole population or a subpopulation, the whole species potentially. Or if it’s just a genotype, a phenotype, that you don’t want, you’re trying to get rid of that and turn it into something else. Get rid of the gregarious desert locust, and force it to be this other thing that you think will work better with human life. And those applications that really sort of live into that extinction ideal—if it’s a native organism, like the desert locust, you’re fiddling with it in its home range—are intrinsically somewhat less attractive to me”
3D	We should compare interventions with GDTs to other interventions in nature that we consider morally permissible	R6: “I suppose one context in which we’d want to put is to look comparatively at the kind of interventions we're very happy to do in nature without any without much notion what the consequences will be. And for you know with perhaps much lesser potential benefits, I mean clear cutting a large forest or something (..), probably changing the environmental, meteorological, all kinds of factors in unpredictable ways. Probably for very questionable goals like replacing them with a large plantation of food stuff. (..) it has some relevance to evaluating the way we should think about this kind of intervention, and we should remember that we intervene all the time”
3E	Nature is not good, and this provides a reason to intervene	R22: “[There are] people who feel that nature is important on a spiritual level and that it [should be] unaffected by humanity as much as possible. (..) I completely disagree because in my view much of nature is—well, nature is amoral and that’s a bit of a problem because when you look at it with a moral lens you see an awful lot of animals suffering. (..) I’m not at all convinced that nature is good”
3F	GDTs do not intervene in a ‘natural’ state of affairs	R5: “A lot of the ethical debate around gene drive has the preconception or the assumption that nature is still in a natural state (..) they fail to recognize that there is a[n] (..) assumption from the beginning: that nature created by whatever force is perfect. And then it's perfect and what we're doing today [in] 2019 is affecting it. But we've been here for a really long time”
	Balancing the value and interests of humans, non-human animals and nature	
3G	Human interests outweigh animal and environmental interest	R26: “I'm big on (..) trying to check my privilege (..). [if] you're a westerner, ecology is allowed to be your biggest concern, versus someone who lives in Africa whose children are dying. And as a human, like, our biggest concerns are human concerns”
3H	The way in which human interests always take precedence should be questioned	R30: "I think we have a very contentious, a very bizarre relationship with nature. (..) [I] think it can be universally agreed upon [that] nature, however you define it, is shrinking and it’s shrinking because we’re ever-expanding. And so the question is: As we ever-expand, what does that mean for us and what does that mean for whoever lives in the remaining nature that still exists? Do we have any obligation being the critter who’s the most exploitative of the planet, the most inconsiderate, the most free-ranging here, and the most volatile and the most detrimental to other species, how do we and do we have an obligation? Is there any kind of moral obligation to take that into account? ”
3I	The interests of humans should not trump the interests of non-human animals and the environment	R19: “I’m seeing, more and more, human beings as part of the whole biosphere and therefore not just having a special claim in a way. Of course I’m a human, so in that sense I can see why, but it seems to me as though humans have been making a special case for their own interests for a very long time, and I don’t know where that’s got us (..) Between the application of (..) island invasive species and malaria, on the surface of it there might seem to be an ethical difference, but in the greater picture of a planet and the fact that we have to change our attitudes to this planet (..), I don’t”
3J	Taking the value and interests of the non-human into account provides conditions for use of GDTs	R32: “It becomes particularly complicated when we’re faced with something like a public-health imperative. (..) How can you say mosquitos are important enough not to save 500,000 lives a year? (..) There must be ways we can uphold both and something like compassion (..) [for both] people who are dying (..) [and] for the environment that could be damaged by making these choices. (..) For example, if you feel the flourishing of both should be supported, then a strategy that has the potential to drive the species to extinction probably doesn’t fit in that model (..). It doesn’t mean there might not be other strategies that could still succeed in reducing malaria transmission through genetic modification of mosquitos”

Other respondents (from different disciplines) disagreed with this view on the role of humans in nature and did not have fundamental problems with interfering in nature (‘interference’ stance). Some of these respondents argued that we intervene in nature all the time, and generally appear to consider it morally permissible to do so. Rather than looking specifically at GDTs, some of these respondents argued we should look comparatively at other interventions in nature that we consider morally permissible. If we consider other drastic interventions in nature morally permissible, it would be inconsistent to object to GDTs on the grounds that these technologies would be used to intentionally change nature (Table 3, Quote 3D). Some of these respondents also criticized opponents’ views on another ground, namely that they have an overly optimistic view of the goodness of nature. According to these respondents, nature is characterized by suffering and pain (as is, for instance, illustrated by the suffering of many wild animals). In their view this suffering provides moral grounds to intervene in nature, rather than to preserve it as it is (Table 3, Quote 3E). Other respondents questioned whether something like a ‘natural’ state of affairs that can be preserved actually exists. These respondents contended we should not see the current distribution of organisms as the ‘natural’ state of affairs which ought to be protected from human influence, as nature has been influenced by humans for millennia (Table 3, Quote 3F). According to these respondents, these in their opinion incorrect views of nature (as either inherently good or untouched and pristine) lead to unjustified conclusions about the impermissibility of using GDTs in nature.

#### Balancing the value and interests of humans, non-human animals and the environment

The positions of respondents about the role humans should take in nature were also related to their opinions about the value and interests of humans, non-human organisms and the environment, and how these should be balanced in decision-making about whether (and if so, under what conditions) to use GDTs. Various respondents argued that human interests outweigh the interests of non-human animals and the environment (Table 3, Quote 3G). Other respondents questioned the way in which human interests always take precedence over the value and interests of non-human animals and entities, and argued the latter are insufficiently taken into account (Table 3, Quote 3H). Several other respondents argued that the interests of humans should not trump the interests of non-human animals and the environment, and that GDTs should thus not be used (Table 3, Quote 3I). Others instead argued that these considerations limited potentially justifiable applications of GDTs to those applications that would achieve great benefits for humans (such as public-health benefits) while minimally affecting non-human animals (Table 3, Quote 3J).

In summary, respondents held different views on what the role of humans in nature should be, whether or not there is a moral reason to preserve the ‘natural’ state of affairs, and how the value and interests of humans, non-human animals and the environment should be balanced. Views on these issues influenced their views on GDTs and contributed to different stances on whether applications of GDTs could be justified, and if so, under what conditions. On these grounds, some respondents considered it permissible to intervene in nature using GDTs (‘interference stance’), whereas others did not (‘non-interference stance’). Natural scientists and policy makers were more inclined to hold the former stance, whereas respondents working within philosophy/ethics, the social sciences or NGOs were somewhat more inclined towards the latter.

## Discussion

As far as we know, this study is the first in-depth interview study in which the moral views of a broad range of GDT experts were investigated. Our analysis sheds light on the considerations that influence the moral views of experts about the permissibility of (applications of) GDTs. Three main themes were identified: (1) how the uncertainty related to GDTs should be approached; (2) the alternatives to which GDTs should be compared and how these alternatives should be weighed; and (3) the role humans should have in nature.

In what follows, we will reflect on the implications and relevance of our empirical study for the debate on GDTs, relate its findings to the broader literature, and identify areas for further research. First, we will reflect on those issues about which experts largely agreed. Subsequently, we will discuss the disagreements that the study identified and underline issues that demand further (normative) reflection. Finally, we will outline some limitations of our study and provide recommendations for future research.

### Common ground

To start, this analysis points to issues about which experts with different moral views on GDTs were in accordance, even if their overall views on the moral permissibility of these technologies differed vastly. First, experts with different moral views identified similar concerns with regard to the existing knowledge gaps for particular gene drive designs and applications, technical hurdles that would need to be overcome, and areas of uncertainty related to translation of results obtained in the laboratory to effects in the wild. Those with fundamentally different views on GDTs thus nonetheless agree that knowledge gaps exist and that more knowledge about particular topics should be obtained.

Second, experts pointed out that it is important to set realistic expectations about the complexities and uncertainties involved in estimating the effects of GDTs, both in terms of the potential benefits and risks. The importance of openness and transparency about uncertainties about both potential benefits and harms have been recognized by various organizations and authors in the GDT field (e.g. [43–45]), yet the results of our study emphasize that GDT experts nonetheless continue to see overhyping of these technologies as a risk. This is a relevant finding since expectations about new and emerging technologies are ‘performative’: they do not merely constitute representations of potential future scenarios, but also contribute to shaping the future, for example by influencing agenda setting and resource mobilization [35, 46–48]. As discussions on hype underline, expectations about emerging technologies can also have concrete undesirable impacts. For GDTs, it has been noted that unrealistic expectations could lead to premature calls for their release [1]. Furthermore, hyping could distort publics’ and communities’ understanding and expectations of these technologies, and potentially lead to a loss of credibility or trust if expectations are not fulfilled [46, 48]. Additionally, unrealistic expectations may divert resources away from alternative strategies that may in fact be better suited to tackle a particular problem. This may be seen as especially problematic in view of concerns about path dependency, the idea that investment in one particular solution to a problem makes it harder to switch to another solution even if it turned out to be superior [12, 46].

For GDT experts, and in particular GDT scientists, it is thus important to balance enthusiasm—which is both understandable and necessary to build momentum and raise funds in any scientific endeavor [49]—with the concomitant responsibility to be open about complexities, uncertainties and knowledge gaps. Moreover, this confirms the importance of obtaining more information to address current knowledge gaps, realistically weighing different alternatives to achieve particular aims [9] and designing adequate evaluation and mitigation plans [1]. Furthermore, it could be valuable to make different visions about GDTs themselves a subject of analysis throughout the process of their development. As the literature on ‘sociotechnical imaginaries’ illustrates, these visions are strongly influenced by broader visions of desirable futures [50]. A critical analysis of these different visions enables a transparent discussion about the plausibility and desirability of the different underlying arguments, premises and imaginations in which they are grounded, and could thereby help provide orientation on GDTs [35, 51].

### Sources of disagreement

For each of the three themes that were identified, there were also fundamental disagreements; whilst experts with different opinions agree on particular (empirical) issues, they disagree about what we should do in light of these issues. In what follows, the sources of these disagreement will be explored in more detail.

#### Disciplinary differences

In previous studies, it has been posited that professionals from different disciplines have different approaches that affect their views on emerging technologies. For example, Ndoh, Cummings and Kuzma [52] describe disciplinary culture as a factor in risk perception. In their study, natural scientists for instance had lower expectations of human and environmental hazards of a synthetic biology case study than social scientists. Amongst other things, these differences may be explained by disciplines’ different epistemological underpinnings and knowledge approaches, which each have their own preoccupations, strengths and weaknesses [53]. These different disciplinary approaches are also tied to different value-based positions [54].

In our study, respondents working in the natural sciences, philosophy/ethics and policy making were somewhat more inclined to have ‘liberal’ stances (that leaned towards deploying GDTs) in relation to the three themes that were identified, whereas respondents working in the social sciences and NGO sector were more inclined to have ‘conservative’ stances (that leaned towards refraining from deployment of GDTs outside the laboratory). Whilst our study was set up with the aim of studying the moral views of a wide range of GDT experts rather than studying the influence of disciplinary cultures on these views, these differences underline the relevance of interdisciplinary collaboration in the development of and decision-making about GDTs, for each discipline can contribute its own insights and perspectives. At the same time, there was also significant variation in stances *within* groups of respondents working in the same discipline, demonstrating that the differences in moral views could not be attributed or reduced to the respondents’ disciplinary cultures. In the following sections, we will therefore get to the heart of these disagreements by investigating the basis for the identified tensions in more detail.

#### Consequences of knowledge gaps and epistemic uncertainty

The first source of disagreement that was identified concerns the consequences of the knowledge gaps and epistemic uncertainty related to GDTs. Epistemic uncertainty is widely recognized as a persistent characteristic of new and emerging technologies in general and of GDTs in particular [1, 3, 10, 21, 45]. As has also been recognized, reducing epistemic uncertainty about the risks of GDTs could paradoxically require an environmental release that itself poses risks^3^ [21, 25], underlining the importance of determining when knowledge gaps can be considered sufficiently resolved to make responsible decisions about specific GDT applications [25, 55].

In our study, some respondents argued the knowledge gaps and uncertainty provide a rationale to proceed with phased testing (potentially including, at some point, field testing) to obtain more data, whereas others argued there should be a moratorium on any application of GDTs outside the laboratory. Whilst both groups of respondents argued that it is important to prevent risks and harms, they operationalized this differently: the latter group contended greater precaution should be taken against the risks and harms associated with the *use* of GDTs (‘risks of intervention’ stance), whereas the former group instead placed greater weight on the opportunity costs associated with *failing to use* GDTs (‘risks of non-intervention’ stance) and argued proceeding with GDT deployment could be acceptable even if uncertainty remained.

The identified stances can also be recognized in broader disputes between those that respectively take a ‘precautionary’ or a ‘proactionary’ stance toward emerging technologies, in which the latter have argued that precaution in one respect often leads to increased risks and harms on other fronts [56–58]. A crucial point of difference between these different parties also relates to who should bear the burden of proof with regards to a technology’s potential to cause harm, with those that consider novel technologies ‘guilty until proven innocent’ versus ‘innocent until proven guilty’ at opposite sides of the spectrum [57]. At the same time, it has also been argued that it could be possible to escape this polarization by distributing the burden of proof [57], explicitly framing precautionary courses of action as precautionary “*with respect to* something” (p. 469) [56], and looking for a middle ground of ‘optimal’ rather than ‘maximum precaution’ [59]. Whilst these proposals may not resolve the dispute between those with different stances, they may nonetheless help to bring points of disagreement more squarely into focus in related discussions. Moreover, these different stances invoke discussion about other related questions such as what constitutes a ‘benefit’ or a ‘risk’ in the first place [45, 60], and how potential benefits and risks should be weighed.

#### Weighing of alternative strategies

The second source of disagreement that was identified in this study concerns the weighing of alternative strategies to confront vector-borne diseases, invasive species and agricultural pests. Whereas many respondents compared GDTs to other ‘downstream’ solutions such as pesticides, other respondents argued ‘upstream’ solutions that tackle these problems at their root should be deployed instead. Some respondents, for instance, stressed that the impact of vector-borne diseases such as malaria is also determined by social and political factors, and argued that deploying GDTs would thus not offer lasting solutions. These different stances were also mentioned in the report of a workshop that identified governance issues and research needs in relation to GDTs [25].

To some degree, these different stances may be attributable to a different understanding of the empirical data on the efficacy of past efforts to confront these problems and the factors that influence future projections of success if these strategies are continued or intensified [12]. To the extent this is the case, making empirical convictions about both ‘downstream’ and ‘upstream’ alternatives to GDTs will further (policy) discussions about GDTs. However, these different stances also point to deeper normative questions about the (im)permissibility of ‘technological fixes’, a recurrent theme in debates about biotechnology [61] that has also received some attention in the debate on GDTs [12, 45, 62]. Such techno-fixes have, amongst others, been critiqued on the ground that they reduce a social problem to a technical problem, which could both perpetuate the underlying problem [1, 62] and result in problematic side-effects [45]. Moreover, it has been argued that techno-fixes are based on mistaken convictions about the inherent progressiveness of science and technologies [61]. At the same time, it has been noted that the comparatively quick and targeted nature of GDTs could nonetheless make them an attractive solution [1], raising relevant questions about whether we ought to take ‘ideal theory’ or ‘non-ideal theory’ (which focuses on what we ought to do in non-ideal circumstances) as a starting point for ethical decision making about GDTs.

#### Intervening in nature

A third normative dispute related to the permissibility of using GDTs to intervene in nature for our aims and benefit. Experts’ views of nature and the role of humans in it, as well as their views on the value and interests of humans, non-human animals and the environment, impacted their moral view on intervening in nature with (particular applications of) GDTs. Views of nature and what is ‘natural’ have been found to influence views on a broad range of emerging technologies [63, 64] and have long since led to debate about the ideal of nature and the (ir)relevance of naturalness as an ethical criterion [9, 65, 66]. As has also been pointed out, people across the world moreover tend to have different views of (the role of humans) nature [45], underlining that it would be highly relevant to study how related perspectives affect non-Western experts’ and publics’ moral views of GDTs.

In the literature about GDTs, various authors and organizations have indeed pointed out that perspectives on the relationship of humans to nature play an important role in the debate about these technologies [1, 30, 45, 67, 68]. Relevant points of contention in this debate include if and on what grounds human independence is valuable in (wild) species and ecosystems [45, 62], if and on what grounds GDTs differ in morally relevant ways from other interactions with non-human nature [45, 67] and if and on what grounds it is permissible to genetically modify non-human organisms to achieve conservation goals rather than changing human behavior to achieve these goals^4^ [62]. As the results of our study imply, stances about the permissibility of intervening in nature in this way also hinge on convictions about the value or moral status of different organisms, what duties we have towards these different entities, and how duties towards different entities should be prioritized in case they conflict. Although these featured less prominently in respondents’ statements, convictions about the value or moral status of holistic entities such as species [13] and ecosystems are likely to be of similar relevance.

For all these different normative disputes, critical analysis and explicit discussion can help to disentangle the complexity of the problems at hand, challenge potentially unwarranted assumptions and enable individuals to develop well-considered judgements on these issues. GDT experts, which actively shape these technologies and the debate about them, may take the stances and considerations outlined in this paper as a starting point for further reflection on their (implicit) views on these matters and how these affect their views on GDTs. At the same time, it is important to realize that genuine value pluralism about many of these issues will remain [69], underlining the need for fair governance and decision-making procedures. Amongst others, important procedural ethical questions relate to who should make decisions, how these decisions should be taken, and when deliberation should be concluded [1, 15–17].

### Limitations and recommendations for future research

Our results should be interpreted in the context of the following limitations. First, the scope of our study was relatively broad. As it was the first large and in-depth interview study on experts’ moral views regarding GDTs, we chose to conduct an exploratory study to allow experts to bring up issues they considered relevant. Although saturation was reached on the codes and themes identified, further research should explore these topics in more depth. Second, any qualitative interview study is prone to interviewer and researcher bias; a different interviewer could have focused their attention on different aspects of the respondents’ answers, and grouped the codes and themes differently. Third, our study represents a subgroup of GDT experts which prominently contributed to the academic and/or policy debates on GDTs. While these experts offered a diverse range of perspectives, they were predominantly employed in the global North. It would be highly relevant to conduct additional qualitative interview studies with experts in other countries to investigate whether there are cultural or otherwise region-dependent differences amongst experts. In particular, it would be relevant to focus on respondents from countries where GDTs may be used to combat vector-borne diseases and/or invasive species, such as Burkina Faso, Ghana, India, Australia and/or New Zealand. Similarly, it would be very relevant to conduct a qualitative study amongst the communities living in areas where GDTs may be deployed. Finally, many of the issues identified in this study warrant a more detailed normative analysis.

## Conclusion

GDTs are developing rapidly and have been proposed as a potential strategy to address several major problems, but have also raised a range of ethical questions. These technologies themselves, the academic debate on the associated ethical questions, and the related policymaking are shaped by experts from different disciplines. This interview study helps to disentangle the polarized debate on GDTs by providing a better understanding of the moral views of GDT experts and elucidating where they agree and disagree. The obtained insights provide valuable stepping-stones for a constructive debate about underlying value conflicts and point to topics that deserve further academic scrutiny. Further evaluation of these and other morally relevant aspects of GDTs should take place in co-production with diverse stakeholders in parallel to the technological development of GDTs. In this way, these considerations can inform the design and implementation of these technologies and facilitate their responsible development.

## Supplementary Information

**Additional file 1**. Interview guide.

## Data Availability

The interview guide is available in the supplementary information. The datasets generated and analyzed during the current study are not publicly available because individual privacy could be compromised; since the gene drive field is relatively small, individual participants could reasonably be identifiable if the full datasets were provided. The individual privacy of the participants is considered of particular importance because the data in question include participants’ political opinions and philosophical beliefs, which are deemed sensitive and get specific protection under the General Data Protection Regulation (GDPR; article 9).

## Abbreviations

COREQ: COnsolidated criteria for REporting Qualitative studies
CRISPR/Cas9: Clustered Regularly Interspaced Palindromic Repeats/CRISPR-associated protein 9
GDTs: Gene drive technologies
NGO: Non-governmental organization

## Footnotes

1 If organisms have an inheritance pattern that can be biased, typically meaning that they can reproduce sexually (70).

2 We will report on the findings related to the procedural ethical aspects of GDTs, i.e. the questions, concerns, and implications that relate to the process of governance of and decision-making about GDTs, in a separate manuscript.

3 As has been recognized by GDT experts in a workshop on gene drive governance and research needs, the use of localized rather than self-sustained GDTs could reduce this complexity (25).

4 In this sense, the third theme is interrelated with the discussion on techno-fixes, which calls the permissibility of doing so into question. Indeed, techno-fixes have also been critiqued on the ground that they derive from a commitment to an anthropocentric conception of the human relationship to the (rest of) nature (59).
