# Supplementary material for: Experts’ moral views on gene drive technologies: a qualitative interview study
Source: BMC Med Ethics. 2021 Mar 8;22:25. doi: 10.1186/s12910-021-00588-5 (PMC7938529; doi:10.1186/s12910-021-00588-5)
Supplement: Supplementary file 1 — Additional file 1. Interview guide. [file 12910_2021_588_MOESM1_ESM.docx]

**Additional file 1** – **Interview guide**

*As specified in the Methods ‘Data collection’ subsection, the interviews consisted of open-ended questions related to potential benefits, risks and broader ethical implications of GDT, stakeholder involvement and governance of GDT. The semi-structured design of the study ensured consistency in a number of topics to be discussed by all participants, while also allowing participants to bring up or emphasize particular new issues they considered relevant. This article reports the interview findings related to what may be classified as the substantive ethical questions, concerns, and implications of GDT. We will report on the findings related to the procedural ethical aspects of GDT in a separate manuscript.*

1. Can you introduce yourself and explain in what way you are involved with or have experience with gene drive technologies?
2. How do you view gene drive technologies based on your experience?
   1. Potential benefits, risks, hazards, ethical implications?
   2. How should we deal with these?
3. How do you view the different potential applications of gene drive technologies (eradicating vector diseases, controlling invasive species, controlling agricultural pests)?
4. How do gene drive technologies relate to alternative strategies to achieve these goals, in your opinion?
5. Various types of gene drives, as well as various gene drive designs are under development. Do you know these different gene drives, and if so, how do you view these?
6. What are, in your opinion, conditions under which gene drive technologies could be used, or limits that should be in place?
7. Who should make decisions about the development and possible use of gene drive technologies? What should, for example, be the role of scientists, the government or governments, and citizens?
8. Do you have experience with current regulation or safety standards for research with (and development of) gene drive technologies? If so, what do you think of the current regulation and safety standards / what should be addressed in such regulations or standards?
   1. Should this be approached in an international context? If so, how?

How should gene drive research develop, as far as you are concerned?

- 1. For example, what would be needed to draw conclusions about whether or not gene drive technologies should be applied and if so, how?

1. Are there topics that have not been addressed that you would still like to discuss?
